# Supplementary material for: Pediatric Primary Hepatic Tumors: Diagnostic Considerations
Source: Diagnostics (Basel). 2021 Feb 18;11(2):333. doi: 10.3390/diagnostics11020333 (PMC7922091; doi:10.3390/diagnostics11020333)
Supplement: Supplementary file 1 [file diagnostics-11-00333-s001.zip › Tables/Supplemental Table 1. Published Cases of Pediatric Hepatic Angiosarcoma in the Literature from 1944 to 2020.docx]

|  | **Author** | **Publication Date** | **Age (Gender)** | **Initial Diagnosis** | **Clinical Presentation** | **Outcome** |
| --- | --- | --- | --- | --- | --- | --- |
| *1* | *Andries and Kaump* | 1944 | Newborn (F) | Multiple malignant hemangiomas | Clinical course unknown  Metastases retroperitoneum, gallbladder and adrenals | Death |
| *2* | *Kauffman et al.* | 1960 | 1 yo (F) | Not documented | Clinical course unknown. Pulmonary metastases. | Death |
| *3* | *Dehner et al.* | 1971 | 6 patients: aged 13 days to 4 yo | Not specified | Clinical course unknown | Death |
| *4* | *Adam et al.* | 1972 | 3 yo (F) | Hepatoblastoma | Abdominal distension, right upper quadrant mass, weight loss, ascites, pitting edema  Labs: anemia | Death |
| *5* | *Sugahara et al.* | 1974 | 15 yo (M) | Angiosarcoma | Clinical course unknown | Death |
| *6* | *Chabalko and Fraumeni* | 1974 | 10 days – 3 years (1F, 1M, 3 unknown) | 1 angiosarcoma  4 unknowns | Clinical course unknown | 2 deaths, 3 unknowns |
| *7* | *Falk et al.* | 1981 | 20 mo (F) | Hepatoblastoma | Abdominal swelling, pica, lung metastases  Arsenic exposure | Death |
| *8* |  |  | 34 mo (F) | Infantile hemangioendothelioma | Asymptomatic with abdominal mass and cutaneous hemangiomas on exam | Death |
| *9* |  |  | 23 mo (F) | Infantile hemangioendothelioma | Abdominal swelling, pica, lung metastases | Death |
| *10* |  |  | 2 mo (F) | Infantile hemangioendothelioma | Abdominal enlargement, cardiac failure | Death |
| *11* | *Kirchner et al.* | 1981 | 4 yo (M) | Hemangioma | Enlarging epigastric mass, anemia. Patient with history of treatment for cutaneous and hepatic hemangiomas from 6 to 10 months of age. | Alive – 2 years post diagnosis with no evidence of disease |
| *12* | *Moazam et al.* | 1982 | 6 mo (F) | Angiosarcoma | Clinical course unknown  Metastases to serosa | Death |
| *13* | *Weinberg and Finegold* | 1983 | 23 mo (F) | Malignant hemangioendothelioma | Clinical course unknown.  Lung and lymphatic metastases | Death |
| *14* |  |  | 15 yo (M) | Angiosarcoma | Clinical course unknown | Death |
| *15* |  |  | 20 mo (F) | Hepatoblastoma | Clinical course unknown  Lung metastases | Death |
| *16* |  |  | 34 mo (F) | Infantile hemangioendothelioma | Clinical course unknown  No metastases | Death |
| *17* |  |  | 23 mo (F) | Infantile hemangioendothelioma | Clinical course unknown  Lung metastases | Death |
| *18* |  |  | 2 mo (F) | Infantile hemangioendothelioma | Heart failure | Death |
| *19* |  |  | 4 yo (F) | Multiple hemangioendotheliomas, Type 1 | History of hemangiomas at 6 months of age. Mass, anemia and angiosarcoma found at 4 years of age. | Alive – 2 years after therapy |
| *20* |  |  | 3 mo (M) | Multiple hemangioendotheliomas, Type 1 | Angiosarcoma replaced liver and ruptured | Death |
| *21* |  |  | 5 yo (M) | Angiosarcoma | Lytic bone lesion | Death |
| *22* |  |  | 4.5 yo (M) | Angiosarcoma | Clinical course unknown | Death |
| *23* | *Noronha* | 1984 | 5 yo (F) | Hemangioma | 1 week history of abdominal pain and vomiting with abdominal mass  Labs: normal liver enzymes initially but then elevated at recurrence, elevated LDH | Alive – 4 months post-op liver resection |
| *24* | *Strate et al.* | 1984 | 3 mo (M) | Infantile hemangiomas | Clinical course unknown | Death |
| *25* | *Alt et al.* | 1985 | 14 mo (M) | Angiosarcoma | Abdominal distension., hepatosplenomegaly, cutaneous hemangioma  Developed ling metastases and splenic angiosarcoma  Labs: thrombocytopenia | Death |
| *26* | *Selby et al.* | 1992 | 1.5 yo (F) | Not documented | Mass, jaundice, URI  Subpleural metastases  Labs: Hgb 5.5 g/dL | Death |
| *27* |  |  | 4 yo (M) | Not documented | Mass for 6 months, jaundice  Adrenal, mesenteric, right renal metastases  Labs: Hgb 3.6 g/dL, TBili 14.6 g/dL, AST 261/IU | Death |
| *28* |  |  | 7 yo (M) | Not documented | Mass, abdominal pain, diarrhea, no weight gain, hemoperitoneum during surgery  Labs: Hgb 9.7 g/dL | Death |
| *29* |  |  | 4 yo (F) | Not documented | Unknown | Unknown |
| *30* |  |  | 2 yo (F) | Not documented | Mass, cutaneous hemangiomas | Unknown |
| *31* |  |  | 3 yo (F) | Not documented | Mass, pallor, fatigue, vomiting  Right pulmonary metastases  Hgb: 8.0 g/dL | Death |
| *32* |  |  | 5 yo (F) | Not documented | Unknown | Death |
| *33* |  |  | 4 yo (M) | Not documented | Mass for three months, jaundice | Death |
| *34* |  |  | 3 yo (F) | Not documented | Unknown | Alive |
| *35* |  |  | 4 yo (M) | Not documented | Mass | Unknown |
| *36* | *Awan et al.* | 1996 | 6 mo (F) | Hemangioendothelioma | Failure to thrive, cutaneous hemangiomata, cardiac murmur, hepatomegaly initially then represented at 3 yo with abdominal distension. Continued to progress with development of pulmonary metastases. | Death |
| *37* |  |  | 3 yo (F) | Hemangioendothelioma | Abdominal distension, hepatomegaly, ascites, peripheral edema. Normal AFP. | Death |
| *38* |  |  | 3 yo (M) | Hemangioendothelioma | Abdominal distension, massive hepatomegaly. Pulmonary metastases present. | Death |
| *39* |  |  | 6 yo (M) | Angiosarcoma | Abdominal distension, massive hepatomegaly.  Congestive heart failure with marked ascites at time of death | Death |
| *40* | *Gunawardena et al.* | 1997 | 3 yo (F) | Angiosarcoma | Fever, decreased appetite, rhinorrhea, abdominal mass on exam  Labs: elevated LDH | Alive |
| *41* | *Valle et al.* | 1998 | 12 yo (F) | Angiosarcoma | Presented with diffuse angiomatosis and extensive angiosarcoma of liver and spleen | Death |
| *42* | *Dimashkieh et al.* | 2004 | 5 yo (F) | Infantile hemangioendothelioma with atypical features | Abdominal mass at presentation  Progressed to have acute abdominal pain with distension, fever, respiratory distress, generalized edema and rapid drop in her hematocrit due to active intraabdominal bleeding. Developed significant coagulopathy and hemorrhagic strokes. | Alive at time of publication however with pulmonary metastases 14 months post-transplant |
| *43* | *Nazir and Pervez* | 2006 | 25 day old (F) | Angiosarcoma | Presented with abdominal distension, respiratory distress, and feeding difficulties found to have marked ascites and edema of lower extremities.  Normal AFP | Death |
| *44* |  |  | 21 day old (F) | Type 2 infantile hemangioendothelioma | Noted to have 2 cutaneous hemangiomas (back and scalp) at birth. On day of life 15, she presented with  respiratory distress, feeding difficulty, and abdominal distension. She developed jaundice, respiratory distress secondary to massive  hepatomegaly, and hemoptysis with eventual cardiorespiratory failure.  Labs: anemia, thrombocytopenia, coagulopathy, normal AFP | Death |
| *45* | *Nord et al.* | 2006 | Newborn (F) | Multiple hemangiomas | Tachypnea at four hours of life. Found to have systolic murmur and hepatomegaly on exam with progressive development of cutaneous infantile hemangiomas. Hepatic lesions identified on day of life four by ultrasound. Progressed to have high output cardiac failure and failure to thrive. | Alive at 13 months of age after orthotopic liver transplant with no evidence of disease |
| *46* | *Geramizadeh et al.* | 2010 | 7 yo (M) | Hemorrhagic hemangioma with atypical imaging features | Abdominal pain, loss of appetite  Mild transaminitis, cancer antigen 125 elevated, AFP normal | Death, but no evidence of disease |
| *47* | *Ackermann et al.* | 2011 | 30 mo (F) | Multiple hepatic hemangiomas | Initially presented with hepatomegaly, cutaneous hemangiomas at 6 weeks of age. Patient had multinodular hemangioma of the liver with initial improvement. At 30 months of age, had diffuse pain and increased hepatomegaly with jaundice, disseminated intravascular coagulation and death. | Death |
| *48* |  |  | 3.5 yo (F) | Multiple hepatic hemangiomas | Presented at birth with cutaneous hemangiomas and developed multiple liver hemangiomas at age 3 months which resolved with treatment at 2 years old. At 3.5 years patient experienced intraperitoneal bleeding and was found to have a large vascular tumor on left lobe of liver. She experienced progression of the tumor with associated abdominal bleeding, jaundice, disseminated intravascular coagulation and death. | Death |
| *49* |  |  | 5 yo (F) | Multiple hepatic hemangiomas | Initially presented with multiple liver hemangiomas, cutaneous hemangiomas at 2 months of age which responded to treatment. At age 5 had progression with biopsy confirmation of angiosarcoma and rapid increase in size of lesions with development of refractory ascites and death from multisystem organ failure | Death |
| *50* |  |  | 2 yo (F) | Type 1 Hemangioendothelioma | Hepatomegaly. 3 months after presentation had rapid increase in size with refractory ascite and pulmonary metastases.  Labs: normal AFP | Death |
| *51* |  |  | 3.5yo (F) | Angiosarcoma | Hepatomegaly. Transplant 1 month after diagnosis.  Labs: normal AFP | Alive – 3 years after liver transplant |
| *52* | *Faria et al.* | 2013 | 3 yo (F) | Angiosarcoma | Not documented | Alive |
| *53* | *Lopez et al.* | 2013 | 20 yo (M) | Hemangioendothelioma | Presented with somnolence, disorientation, sleep disturbances, upper abdominal pain, increased abdominal girth. Of note, he had an occupational exposure to vinyl chloride.  Exam notable for altered level of consciousness, mucocutaneous jaundice, ascites, hepatosplenomegaly and lower extremity edema.  Labs: thrombocytopenia, coagulopathy, transaminitis, extreme direct hyperbilirubinemia | Death |
| *54* | *Jeng et al.* | 2014 | 5 mo (M) | Infantile hemangioma | Presented at 5 months of age with emesis. Found to have multiple hepatic infantile hemangiomas which were treated and followed.  At 47 months presented with abdominal distension, anemia and progression of hepatic lesions. Pulmonary nodules noted at 50 months of age. | Death |
| *55* | *Olson et al.* | 2014 | 17 yo (M) | Not documented | Patient with severe dyskeratosis congenita. Progressively worsening cholestatic liver dysfunction with hyperbilirubinemia. | Death |
| *56* | *Xue et al.* | 2014 | 5 yo (F) | Infantile hepatic hemangioma | Abdominal distension, early satiety, shortness of breath which worsened over a one-month period. During admission had worsening respiratory status, intermittent right shoulder pain, constipation and anorexia.  Labs: hypothyroidism, mild coagulopathy, | Alive – no evidence of disease 27 months post-transplant |
| *57* | *Potanos et al.* | 2015 | 3 yo (F) | Angiosarcoma | Right sided abdominal pain and general malaise, tachypnea, hepatomegaly.  No evidence of metastatic disease at presentation.  Normal AFP. | Alive – 6 years post therapy with no tumor recurrence |
| *58* | *Kamath et al.* | 2016 | 2 yo (M) | Infantile hemangioendothelioma | Progressively worsening abdominal distension which started at age 5 months with dyspnea with activity. | Lost to follow-up |
| *59* | *Grassia et al.* | 2017 | 3 yo (M) | Infantile hemangioma | Abdominal distension, poor feeding.  Pulmonary metastases at presentation  Patient had transformation from IH to low-grade and then fulminant angiosarcoma | Death |
| *60* |  |  | 2.5 weeks (F) | Vascular tumor versus angiosarcoma | Liver lesions with brain, skin, bone lesions representing metastases | Death |
| *61* |  |  | 3 yo (M) | GLUT-1+ hemangioma with islands of angiosarcoma | Abdominal distension  Pulmonary metastases at presentation | Death |
| *62* |  |  | 4 yo (F) | Infantile hemangioendothelioma (GLUT-1+) with high proliferation | Abdominal distension, pain  No metastatic disease at presentation | Alive – 5.5 years post transplant, disease free |
| *63* |  |  | 2 yo (F) | Transformation of an infantile hemangioma with high-grade GLUT-1+ angiosarcoma | Liver lesions soon after birth, 2 years later presented with increased abdominal girth, found to have new liver lesions (transformation of infantile hemangioma to high grade GLUT-1+ angiosarcoma) | Death |
| *64* |  |  | 4 yo (F) | Liver mass: infantile hemangioendothelioma  Scalp Lesion: angiosarcoma | Age 2: presented with liver mass  Age 4: presented with bilateral leg pain and bony mass of scalp representing metastatic hepatic angiosarcoma | Death |
| *65* |  |  | 3 yo (F) | Infantile  hemangioma with  small foci of hypercellularity/  cytologic  atypia/increased  mitotic activity that  may represent  pre-malignant  transformation but  not angiosarcoma | Abdominal distension  Pulmonary metastases at presentation | Alive – 3.5 years post transplant |
|  |  |  | 3 yo (M) | Hemangioma | Abdominal pain and distension | Death |
| *66* | *McLean et al.* | 2017 | 6 wk (F) | Angiosarcoma | Blood tinged emesis, lethargy, decreased oral intake, abdominal distension, pallor. Later developed abdominal compartment syndrome from intratumoral hemorrhage.  Labs: anemia (Hgb 4.9), thrombocytopenia, mild coagulopathy, transaminitis, direct hyperbilirubinemia, hyperthyroidism | Death |
| *67* | *Pilbeam et al.* | 2019 | 2 yo | Angiosarcoma | Asymptomatic RUQ mass with mild abdominal distension  Labs: mild microcytic anemia, elevated GGT, normal AFP | Alive – after 24 months post liver transplant with no evidence of disease |
