# Supplementary material for: Pediatric Primary Hepatic Tumors: Diagnostic Considerations
Source: Diagnostics (Basel). 2021 Feb 18;11(2):333. doi: 10.3390/diagnostics11020333 (PMC7922091; doi:10.3390/diagnostics11020333)
Supplement: Supplementary file 1 [file diagnostics-11-00333-s001.zip › Tables/Supplemental Table 2.docx]

| **Tumor** | **Benign or Malignant** | **Age Group** | **Clinical Characteristics/Complications** | **Associated Syndromes/Risk Factors** | **Laboratory Findings** | **Immunohistochemistry** |
| --- | --- | --- | --- | --- | --- | --- |
| **Hemangioma** | Benign | Neonates and Infants | **Focal**: hydrops fetalis, cardiac failure  **Multifocal, Diffuse**: may lead to high output cardiac failure, abdominal compartment syndrome | Prematurity  Low birth weight  Positive family history  **Multifocal, Diffuse**: may be associated with cutaneous hemangiomas | **Focal**: anemia, mild thrombocytopenia  Elevated AFP at birth (should downtrend), normal thyroid studies  **Multifocal/Diffuse:** consumptive hypothyroidism | **Focal**: negative GLUT-1  **Multifocal/Diffuse**: positive GLUT-1 |
| **Mesenchymal Hamartoma** | Benign | Neonates, Infants and Young Children (<2 yo) | Neonatal: fetal hydrops, maternal toxemia, preterm labor and fetal demise  Infants: often asymptomatic, abdominal pain, fatigue, fever | Beckwith-Wiedemann Syndrome  DICER1 syndrome | AFP normal or elevated  GGT mildly elevated | Immunoreactivity to cytokeratin, vimentin, smooth muscle actin, α-1 antitrypsin and desmin |
| **Focal Nodular Hyperplasia** | Benign | Childhood  (median age 8.7 years) | Most commonly asymptomatic  If symptomatic, most common presentation is abdominal pain  Mass effect can cause portal hypertension | Prior history of malignancy: extrahepatic solid tumors, hematopoietic stem cell transplant recipients, exposure to alkylating agents, liver radiotherapy  Underlying liver diseases: congenital or surgical portosystemic Shunts, biliary atresia | Normal AFP, may be elevated in infants | Overexpression of glutamine synthetase in a “map-like” distribution |
| **Hepatocellular Adenoma** | Benign | Adolescence | Mostly asymptomatic  Abdominal pain  25% undergo rupture and subsequent intratumoral or intraperitoneal hemorrhage | Estrogen exposure  Androgen exposure  Glycogen Storage Disease (Types I, III, IV)  Congenital Portosystemic Shunts (extrahepatic)  Prior history of malignancy  Fanconi Anemia  History of Fontan Procedure Hurler Syndrome  Familial Adenomatous Polyposis  Maturity Onset Diabetes of the Young Type 3  Galactosemia  Immunodeficiency  Alagille Syndrome | Normal AFP | **HHCA**: decreased or absent LFABP immunostaining  **IHCA**: positive CRP, positive serum amyloid A  **bHCA**: diffuse and strong expression of glutamine synthetase, nuclear positivity for beta-catenin  **shHCA**: positive Prostaglandin D2 synthase  **uHCA**: none |
| **Hepatoblastoma** | Malignant | Neonates, Infants and Young Children (< 4 yo) | Neonates: acute onset, abdominal mass, respiratory distress and failure, tumor rupture during vaginal delivery  Infants/Young Children: non-specific, abdominal mass, anorexia, pain, fatigue, weight loss, isosexual precocity (males < 3yo) | Prematurity  Low Birth Weight  Parental Tobacco Use  Beckwith-Wiedemann Syndrome  Trisomy 18  Hemihypertrophy  Intestinal polyposis | Elevated AFP  Can present with marked thrombocytosis | INI-1 positive  Positive staining for alpha feto-protein, glypican 3, beta-catenin, glutamine synthetase, vimentin, cytokeratin, Hep-Par1 |
| **Malignant Rhabdoid** | Malignant | Infants and Young Children (median age 11-18 months) | Fever, abdominal distension, abdominal pain, decreased oral intake and emesis  More frequently presents with spontaneous tumor rupture (compared to HB) | None reported | Normal or mildly elevated AFP | INI-1 negative |
| **Hepatic Angiosarcoma** | Malignant | Infants, Young Children (median Age 40 months) | Neonates, Infants: rapid onset abdominal distension leading to abdominal compartment syndrome, hyperthyroidism reported  Younger Children: abdominal mass which may be associated with abdominal pain, emesis, jaundice and/or respiratory distress (pulmonary metastasis common) | History of infantile hepatic hemangiomas | Normal AFP  Consumptive coagulopathy | Positive for endothelial cell markers (CD31 and CD34) |
| **Hepatocellular Carcinoma** | Malignant | Adolescence | Abdominal mass, abdominal pain, cachexia, jaundice  Patients with underlying cirrhosis: ascites, splenomegaly, variceal bleeding, spider naevi, clubbing and encephalopathy | Hepatitis B  Hereditary Tyrosinemia  Post-Fontan procedure  Progressive Familial Intrahepatic Cholestasis (most commonly PFIC-2)  Glycogen Storage Diseases (most commonly Type 1a)  Biliary Atresia  Budd Chiari  Alpha-1 Anti-Trypsin Disorder  Congenital Extrahepatic Portosystemic Shunts | Elevated AFP (except in Fibrolamellar subtype where AFP is normal) | NFL-HCC: epithelial cell adhesion molecule, CK-19, Glypican-3, p53  FL-HCC: CK-7 |
| **Undifferentiated Embryonal Sarcoma** | Malignant | Ages 6-10 | Abdominal mass, abdominal pain, fever without jaundice | Unknown | Normal AFP  Mild leukocytosis or leukopenia, anemia, hypoalbuminemia, mildly elevated transaminases and inflammatory markers | IHC is non specific and non diagnostic. Will have expression of fat, muscle, histiocytic and epithelial markers, consistent with its mesenchymal origin |
